# The Relationship Between Dysfunctional Voiding Symptom Score, Functional Bladder Capacity, and Bladder Wall Thickness on Ultrasonography in Children With Primary Nocturnal Enuresis

**DOI:** 10.7759/cureus.118003

**Published:** 2026-10-16

**Authors:** Tsuyoshi Furuno, Takeya Kitta, Haruka Higuchi, Yoko Nishimura, Katsuki Miura, Kaname Ameda, Hidehiro Kakizaki

**Affiliations:** 1 Urology, Hokkaido Memorial Hospital of Urology, Sapporo, JPN

**Keywords:** bladder wall thickness, dysfunctional voiding symptom score (dvss), functional bladder capacity, pediatric urology, primary nocturnal enuresis, ultrasonography (us)

## Abstract

Introduction

This study aimed to evaluate the relationship between self-reported daytime lower urinary tract symptoms (LUTS), Dysfunctional Voiding Symptom Score (DVSS), functional bladder capacity (FBC), and ultrasonographic bladder wall thickness (BWT) in children with primary nocturnal enuresis.

Methods

We evaluated 145 children (98 boys and 47 girls) aged 6 to 10 years diagnosed with primary nocturnal enuresis at our institution between 2020 and 2022. The relationship between self-reported LUTS, DVSS, and BWT was analyzed. Additionally, FBC was measured and the correlation between BWT and FBC was investigated.

Results

Enuresis frequency ranged from one to seven days per week. Of 145 children, 125 (86%) had enuresis four or more days per week. Eighty-one children had no self-reported LUTS. Among those without self-reported LUTS, DVSS daytime incontinence scores of ≥1 (items 1 and 2) were observed in five children (6%), whereas holding maneuvers (item 6) and urgency (item 7) were reported in 22 (27%) and 25 children (31%), respectively. In children with self-reported LUTS, items 1, 2, 6, and 7 were scored ≥1 in 35 (55%), 25 (39%), 38 (59%), and 50 children (78%), respectively. When monosymptomatic nocturnal enuresis (MNE) was defined as the absence of both self-reported LUTS and DVSS voiding items, 46 children were classified as having MNE and 99 as having non-monosymptomatic nocturnal enuresis (NMNE). No significant sex differences were observed between the MNE and NMNE groups. The median BWT was significantly greater in the NMNE group than in the MNE group (4.1 vs. 3.1 mm, p < 0.01). Using a BWT cut-off value of 3 mm to detect NMNE yielded a sensitivity of 86% and a specificity of 48%. FBC was significantly larger in the MNE group than in the NMNE group (median, 150 mL vs. 115 mL; p = 0.024). When the median FBC of the MNE group (150 mL) was used as a threshold, BWT was significantly greater in children with FBC ≤150 mL (n = 40) than in those with FBC >150 mL (n = 15) (median, 4.1 mm vs. 3.2 mm; p = 0.021).

Conclusions

Daytime lower urinary tract symptoms were more accurately assessed by the DVSS than by self-reported symptoms. A structured assessment using the DVSS may help identify unrecognized LUTS and improve the differentiation between MNE and NMNE. Children with NMNE, as defined by the DVSS, and those with smaller FBC demonstrated greater ultrasonographic BWT. These findings suggest that in addition to FBC, BWT could serve as a valuable index of bladder storage function and may assist in treatment selection and predicting therapeutic outcomes.

## Introduction

Nocturnal enuresis (NE) in children is defined as intermittent urinary incontinence occurring during sleep [1]. The prevalence rate ranges from 5% to 13% in school-aged children [2]. NE is classified into monosymptomatic nocturnal enuresis (MNE), defined as the absence of daytime lower urinary tract symptoms (LUTS), and non-monosymptomatic nocturnal enuresis (NMNE), defined as the presence of LUTS. Current guidelines recommend different management strategies for each enuresis type [1].

However, the reported prevalence of LUTS in children with NE varies widely. Ata et al. reported that the use of different questionnaires contributes to these varying rates [3]. Indeed, children or their parents may underrecognize LUTS. Therefore, the dysfunctional voiding symptom score (DVSS) is widely used as a standardized reporting system to evaluate LUTS in children [4].

Recently, the relationship between ultrasonographic bladder wall thickness (BWT), enuresis type, and urodynamic findings was investigated [5-7]. However, in these prior reports, enuresis type was not determined using scoring systems.

Regarding functional bladder capacity (FBC), Telli et al. reported no significant difference in FBC between enuresis types [5], whereas Charalampous et al. reported smaller FBC in the NMNE group [6]. Ata et al. also reported that FBC was smaller in the NMNE group diagnosed using the Dysfunctional Voiding and Incontinence Scoring System (DVISS) [3]. These findings indicate that the method used to detect LUTS is important for accurate evaluation of the relationship between enuresis type and FBC. Although the DVISS has a slightly higher diagnostic accuracy than the DVSS, the DVSS has an officially validated Japanese version [8,9].

Therefore, this study aimed to evaluate differences in LUTS detection between self-reporting and the DVSS. Furthermore, we statistically analyzed the relationship between LUTS, ultrasonographic BWT, and FBC in children with primary nocturnal enuresis (PNE).

## Materials and methods

This retrospective study was approved by the Ethics Committee of the Japanese Urological Association (No. 2025-01). Informed consent was obtained through an opt-out procedure, in accordance with ethical standards.

Children aged 6 to 10 years with PNE who visited our institution between 2020 and 2022 were included in this study. Children with a recent infection or an already diagnosed neurogenic or physical disorder were excluded. We retrospectively reviewed their medical records.

Children (or their parents) who reported daytime urinary symptoms during the initial history-taking were defined as having self-reported LUTS. This history-taking included questions on LUTS. We additionally asked children (or their parents) with PNE to complete the DVSS after the initial history-taking. We diagnosed children as having DVSS-based LUTS when at least one of the DVSS voiding items (items 1 and 2: daytime incontinence, item 6: holding maneuvers, item 7: urgency) was ≥1. MNE was defined as the absence of both self-reported and DVSS-based LUTS, and the remaining cases were classified as NMNE, in accordance with the criteria established by the International Children’s Continence Society [1].

FBC was defined as the largest voided volume in several measurements at home. Successfully measured and collected data were included in the analysis.

Ultrasonographic evaluations were performed by four radiographers specializing in urological examination who were blinded to all clinical information except for the diagnosis of enuresis. Ultrasonography (US) was performed using the Aplio 400 system with a 3.5 MHz convex probe (Toshiba Medical Systems, Japan). BWT measurement was performed when children reported a moderate sensation of bladder fullness. BWT was assessed by measuring 2 to 3 points of the bladder lateral wall on axial images, and the maximum value among them was adopted.

Statistical analysis

We statistically analyzed the relationship between DVSS-based enuresis type, FBC, and BWT on US. Differences in findings between the two groups were statistically analyzed using non-parametric tests (Wilcoxon rank-sum test and Fisher's exact test). A p-value of less than 0.05 was considered statistically significant. The Akaike Information Criterion (AIC) was used to compare the model fit between the two groups. When the difference in AIC between models was ≥2, it was considered to have a significant difference, and the model with the smaller AIC was considered superior. The area under the receiver operating characteristic (ROC) curve was used to determine the cut-off values for BWT and enuresis type. Missing values were handled statistically using pairwise deletion. R software version 4.6.0 (R Foundation for Statistical Computing, Vienna, Austria) was used for statistical analysis.

## Results

A total of 145 children (98 boys and 47 girls; median age, 8.0 years) were included in this study. Their enuresis frequency ranged from 1 to 7 days per week. Of 145 children, 125 (86%) had enuresis four or more days per week, and 81 (56%) had enuresis daily. Thirteen children had already been diagnosed with neurodevelopmental disorders. Asymptomatic hydronephrosis (Society for Fetal Urology grade I-II) was detected in 10 children, who were followed up at our institution. Self-reported LUTS were present in 45 of 98 boys (46%) and 19 of 47 girls (40%), with no significant difference between boys and girls (p = 0.594). Among children without self-reported LUTS, DVSS daytime incontinence (items 1 and 2) was scored ≥1 in 5 (6%), holding maneuvers (item 6) and urgency (item 7) were scored ≥1 in 22 (27%) and 25 (31%), respectively. Among children with self-reported LUTS, items 1, 2, 6, and 7 were scored ≥1 in 35 (55%), 25 (39%), 38 (59%), and 50 (78%), respectively (Table 1).

**Table 1:** Self-reported LUTS and DVSS Item 1: wetting; Item 2: daytime incontinence; Item 6: holding maneuvers; Item 7: urgency Missing data in items 6 and 7 (one point each) were statistically handled using pairwise deletion. LUTS: lower urinary tract symptoms; DVSS: dysfunctional voiding symptom score

	All	Self-reported LUTS = no	Self-reported LUTS = yes
Number	145	81	64
Sex	Boys 98 (67%)	Boys 53 (65%)	Boys 45 (70%)
DVSS	-	-	-
Item 1 = 0	106	77	29
Item 1 = 1	16	3	13
Item 1 = 2	11	1	10
Item 1 = 3	12	0	12
DVSS	-	-	-
Item 2 = 0	119	80	39
Item 2 = 1	13	0	13
Item 2 = 2	9	1	8
Item 2 = 3	4	0	4
DVSS	-	-	-
Item 6 = 0	84	58	26
Item 6 = 1	30	15	15
Item 6 = 2	18	4	14
Item 6 = 3	12	3	9
DVSS	-	-	-
Item 7 = 0	69	56	13
Item 7 = 1	38	18	20
Item 7 = 2	18	4	14
Item 7 = 3	19	3	16

Of 81 children without self-reported LUTS, 35 were identified as having LUTS based on the DVSS. A total of 96 children had at least one item of ≥1 score (61 with self-reported LUTS and 35 without).

We defined MNE as the absence of both self-reported and DVSS-based LUTS. Children with DVSS-based LUTS and three children with only self-reported LUTS were classified as having NMNE. Consequently, the cohort was divided into the NMNE group (n = 99) and the MNE group (n = 46), with no significant difference in the distribution between boys and girls (Table 2).

**Table 2:** Enuresis type and bladder wall thickness P-values of less than 0.05 were considered statistically significant. NMNE: non-monosymptomatic nocturnal enuresis; MNE: monosymptomatic nocturnal enuresis; BWT: bladder wall thickness

	All	NMNE	MNE	P value
Number	145	99	46	
Sex	Boys 98 (67%)	Boys 72 (73%)	Boys 26 (57%)	P = 0.059
BWT	Median 3.7 mm (1.5–7.3 mm)	Median 4.1 mm (1.6–7.3 mm)	Median 3.1 mm (1.5–5.7 mm)	P < 0.01*

The BWT was significantly greater in the NMNE group (median, 4.1 mm; range, 1.6-7.3 mm) than in the MNE group (median, 3.1 mm; range, 1.5-5.7 mm) (Table 2). The BWT in children with self-reported LUTS (median, 4.2 mm; range, 1.6-7.3 mm) was also significantly greater than that in those without self-reported LUTS (median, 3.3 mm; range, 1.5-6.8 mm; p<0.01). However, the DVSS-based enuresis classification (AIC, 448.2) separated BWT more accurately than the enuresis classification based on self-reported LUTS (AIC, 456.9).

The area under the ROC curve for distinguishing enuresis type by BWT was 0.752 (95% confidence interval, 0.665-0.839) with the cut-off value of 3.4 mm. Considering clinical utility and previously reported values [10], we used a cut-off value of 3 mm to classify children into two groups based on BWT. The proportion of NMNE was significantly higher in the group with BWT > 3 mm than in the other group (77% vs. 38%, p < 0.01). For the detection of NMNE by BWT, the sensitivity was 86% and the specificity was 48%.

FBC was measured in 55 children. There was no significant difference in the proportion of MNE/NMNE ratio between FBC-measured and unmeasured groups (31% / 69% vs. 32% / 68%, p = 1.0), nor was there a significant difference in median age (8.0 vs. 8.0 years, p = 0.214). The median FBC was 120 mL (range, 50 - 360 mL). FBC was significantly larger in the MNE group than in the NMNE group (median, 150 mL vs. 115 mL; p = 0.024). When the median FBC of the MNE group (150 mL) was used as a cut-off value, the BWT was significantly greater in the group with FBC of ≤150 mL (n = 40) than in the group with FBC of >150 mL (n = 15) (median, 4.1 mm vs. 3.2 mm; p = 0.021).

## Discussion

The DVSS is widely used as a standardized scoring system for dysfunctional voiding symptoms in children. Although several scoring systems exist, Altan et al. reported that these questionnaires were equivalent for evaluating treatment response, despite the DVISS having higher diagnostic accuracy [9]. The DVSS has officially validated versions in Japanese and other languages [4,8]. In this study, self-reported LUTS underestimated urgency and holding maneuvers compared with the DVSS. Approximately 30% of children without self-reported LUTS exhibited urgency and/or holding maneuvers. The rate of NMNE was higher when we adopted DVSS-based LUTS. Ata et al. reported similar results using DVISS-based LUTS, and recent studies have revealed higher NMNE rates than those in previous reports [3]. The results of the present study suggest that most children and parents fail to recognize urgency or holding maneuvers as LUTS. In addition, the DVSS-based enuresis classification separated BWT more accurately than the enuresis classification based on self-reported LUTS. These findings indicate that appropriate scoring systems are necessary for the accurate evaluation of LUTS, and that enuresis classification should be made by using scoring systems of LUTS.

Previous studies showed that the BWT of the NMNE group was greater than that of the MNE group [5,6]. Consistent with the previous studies, the present study also showed that BWT was greater in the NMNE group than in the MNE group. In the present study, a BWT cut-off value of 3 mm predicted NMNE with a high positive predictive value. Although cut-off values can be affected by examination settings and ultrasonographic systems, previous reports have also proposed approximately 3 mm as a cut-off value [5,11].

Several studies have evaluated bladder condition by measuring both bladder capacity and BWT to calculate an index value [7,10]. However, increasing the number of measurement parameters might complicate the procedure and potentially increase measurement errors. In the present study, evaluation using BWT alone demonstrated a high positive predictive value, suggesting that it is simple and highly useful in clinical practice. Typically, management of PNE is based on enuresis type [12,13]; thus, BWT is considered useful for supporting questionnaire-based assessments.

Detrusor overactivity (DO) has been reported to be more prevalent in patients with a thickened bladder wall [6,7]. Sreedhar et al. reported that, in severe PNE, patients with a thickened bladder wall exhibit a higher frequency of DO and less favorable treatment outcomes [10]. Similarly, Yeung et al. reported the presence of DO and decreased bladder capacity during sleep in children with severe PNE [14]. Detecting DO during the urine storage phase requires invasive urodynamic studies; therefore, estimating DO by ultrasonographic measurement of BWT seems to have high clinical utility.

In the present study, FBC was measured only in 55 out of 145 children (38%). However, there was no difference in age or MNE/NMNE ratio between the FBC-measured and unmeasured groups, suggesting that the FBC measured in the 38% of the patients can be considered to reflect the data of the overall study population. In the present study, the median FBC was 120 mL and appears to be low for this age group. Rushton et al. reported that FBC in children with PNE was smaller than the estimated normal capacity [15]. Thus, we believe that the small FBC in the present study is a characteristic of this cohort. Furthermore, Ata et al. reported that FBC is decreased in children with NMNE evaluated using the DVISS [3], whereas Telli et al. reported no significant difference in FBC between children with MNE and NMNE [5]. In the present study, FBC was significantly decreased in the NMNE group, which is consistent with previous findings based on questionnaires. Further investigations using validated questionnaires are required to clarify the relationship between enuresis type and FBC.

Previous studies reported that the efficacy of desmopressin is diminished in children with small FBC [15,16] and in children with thickened BWT on US [17]. In the present study, BWT was significantly increased in cases with smaller FBC. Because it may be difficult for some patients to measure FBC at home owing to their home environment, BWT might be a useful alternative objective index for predicting treatment efficacy. 

Limitations

The present study has several limitations. First, although there was no difference in age or MNE/NMNE ratio between the FBC-measured and unmeasured groups, FBC was successfully evaluated in 55 of 145 patients, introducing the possibility of selection bias. Second, BWT was measured when children reported a moderate desire to void. Although strict bladder volume monitoring was not performed, we consider this protocol to be a non-invasive and feasible approach for daily outpatient examinations. Finally, this study classified children without self-reported LUTS as NMNE when the DVSS score was positive, but treatment outcomes were not evaluated, suggesting that further studies are warranted.

## Conclusions

Daytime lower urinary tract symptoms were more accurately assessed by the DVSS than by self-reporting. A structured assessment using the DVSS may help identify unrecognized LUTS and improve the differentiation between MNE and NMNE. Children with NMNE, as defined by the DVSS, and those with smaller FBC demonstrated greater ultrasonographic BWT. When a BWT cut-off value of 3 mm was used to detect NMNE, the sensitivity was 86% and the specificity was 48%. These findings suggest that BWT could serve as a valuable index of bladder storage function and may assist in treatment selection and predicting therapeutic outcomes.
